# Influence of the anionic structure and central atom of a cation on the properties of LCST-type draw solutes for forward osmosis

**DOI:** 10.1039/d2ra05131a

**Published:** 2022-10-13

**Authors:** Yeonsu Cho, Hyo Kang

**Affiliations:** BK-21 Four Graduate Program, Department of Chemical Engineering, Dong-A University 37 Nakdong-Daero 550 Beon-Gil, Saha-Gu Busan 49315 Republic of Korea hkang@dau.ac.kr

## Abstract

Thermo-responsive ionic compounds were synthesized to examine if they have a powerful ability to draw solutes for forward osmosis (FO). The investigated compounds were tetrabutylammonium benzenesulfonate, tetrabutylphosphonium benzenesulfonate, tetrabutylammonium 2-naphthalenesulfonate, and tetrabutylphosphonium 2-naphthalenesulfonate (abbreviated as [N_4444_][BS], [P_4444_][BS], [N_4444_][NS], and [P_4444_][NS]). The lower critical solution temperature (LCST) characteristics of the materials that formed the monocyclic aromatic compound [BS] were not confirmed; however, the LCSTs of others that formed the bicyclic aromatic compound [NS] were confirmed to be approximately 37 °C ([N_4444_][NS]) and 19 °C ([P_4444_][NS]) at 20 wt% in aqueous solutions; this is valued in reducing the energy required for recovery of the draw solute. In addition, it suggests that ammonium-based ionic compounds have a higher recovery temperature than phosphonium-based ionic compounds. When an active layer was oriented to a draw solution (AL-DS mode) and using 20 wt% aqueous [N_4444_][NS] draw solution at room temperature, water and reverse solute fluxes were about 3.07 LMH and 0.58 gMH, respectively. Thus, this is the first study to investigate structural transformations of the anion and central atom of the cation and to examine prospective draw solutes of the FO system in this series.

## Abstract 2

Thermo-responsive ionic compounds having lower critical solution temperature were utilized as a draw solute for eco-sustainable forward osmosis.

## Graphical abstract

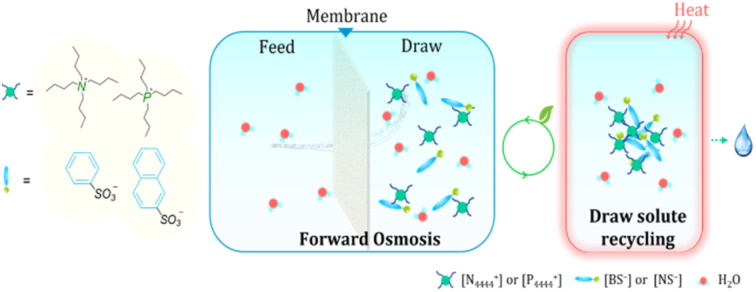

## 1. Introduction

Rapid population growth, rapid industrialization, and limited freshwater resources have led to serious concerns regarding energy crises and the scarcity of clean water worldwide. Potable water supplies and the development of energy sources are co-dependent and have emerged as crucial requirements in our community. Accordingly, various water treatment technologies have been developed, and their commercial feasibility has been demonstrated by categorizing them as distillation,^1^ membrane separation,^2,3^ adsorption,^4^ coagulation, flocculation,^5^ and biological processes.^6^ Among them, membrane desalination technology has been referred to as a distinguished methodology, which ameliorates the aforementioned problems owing to its lower energy consumption and higher efficiency. The method is also advantageous in terms of its selectivity, scalability, simple procedure, and facile operation.^7–9^ As an emerging process, forward osmosis (FO) does not require external pressure to operate this osmosis system owing to a membrane-based process. To drive this process, it relies solely on the natural energy of osmotic gradient differences between the draw and feeds solution, which are on either side of a semipermeable membrane, thus demonstrating higher energy efficiency.^10–13^ FO is a two-step process that consists of osmotically diluting the concentrated draw solute and re-concentrating the dilutive draw solute. In the first step, spontaneous movement of water occurred across the FO membrane. The second step involves regenerating the diluted draw solute to its initial state using an external stimulus. However, the FO process still presents some challenging issues that need to be addressed or improved for the commercialization of the process.^14,15^ The primary impediment to the FO technology is the recovery and reuse of draw solutes in the second step, which is considered to be a potentially energy-intensive process. Therefore, it is necessary to discover a draw solute for being eco-friendly, which can easily be separated from water in the second step and can bring about a great osmotic pressure in the water permeation step.^11,16^

Several researchers have found a variety of draw solutes that can come in two primary categories: non-stimuli-responsive draw solutes and stimuli-responsive draw solutes. Non-stimuli-responsive draw solutes include a myriad of polymers,^17^ gases or volatile compounds,^18^ inorganic compounds,^19,20^ organic compounds such as nutrient compounds,^21–23^ and organic salts.^24^ However, to date, none of these classes of draw solutes can be considered as an excellent choice or a perfect fit; moreover, non-stimuli-responsive draw solutes can render the overall process costly. This is because no noticeable change occurs in their water affinity after stimulation, which necessitates high energy in the draw solute recovery step. Stimuli-responsive draw solutes are generally closer to this target, thereby facilitating the ease of regeneration compared with non-stimuli-responsive draw solutes.^25^ Stimuli-responsive draw solutes can be inferred as compounds with water affinities that can undergo instantaneous changes in response to external stimuli such as magnetic;^26,27^ electric field;^28^ changes in temperature,^29^ pH,^30^ gas,^31^ salt,^32^ and light.^33^

A thermo-responsive property is attractive to draw solute application when separating the draw solution into pure water and draw solute owing to its simplicity and the possibility of utilizing heat sources, such as geothermal, solar thermal energy, and low-grade waste heat from industrial sources.^11^ The thermo-responsive material has various forms in forward osmosis field as draw solute, including polymers,^34–36^ magnetic nanoparticles,^37–39^ hydrogels,^40–43^ and ionic liquids (ILs).^44–48^ In particular, the study of the structures and thermo-responsive properties of ILs has grown exponentially over the past few decades. ILs constitute appropriate draw solutes owing to their easy recovery processes, continuous recycling, and sufficient osmotic pressures generated by the ionic groups, which result in greater water flux. ILs have attracted considerable research interest owing to plentiful structural possibilities, which can be realized by the tweak in the cation–anion combination.^49,50^ In addition, ILs and water can form a homogenous solution and can exhibit unique phase behavior based on the temperature, resulting in a solubility limit. In general, two classes of IL–water mixtures exist and are known as the lower critical solution temperature (LCST) and upper critical solution temperature (UCST) classes.^51,52^ In the phase separation of the LCST-type, the mixture is miscible at low temperature and becomes immiscible above the LCST point, whereas in the phase separation of the UCST-type, the mixture becomes immiscible at low temperature and becomes miscible above the UCST point.^53^ The result of the phase separation is the formation of IL-rich- and water-rich phases. A recent study reported the application of a series of thermo-responsive IL materials to draw solutes. As reported by Liu *et al.*,^47^ LCST-type ILs, including monocationic tetrabutylphosphonium hydrogen maleate (P1Mal), monocationic tetrabutylphosphonium *p*-toluenesulfonate (P1TSO), dicationic tetrabutylphosphonium *p*-toluenesulfonate (P2TSO), and dicationic tetrabutylphosphonium trimethylbenzenesulfonate (P2TMBS), have been investigated to see whether it is possible to potentially act as draw solutes for FO. Although they are potentially suitable for use as draw solutes, research on thermo-responsive IL draw solutes is still limited, and thus, continued search for other effective thermo-responsive ILs and systematic investigations are needed to study their applicability in FO. Based on the abovementioned findings, an ionic compound structure consisting of monocationic and monoanionic forms capable of inducing water permeation appears to be advantageous for efficient FO performance. Additionally, to improve the energy efficiency in the second step, a suitable thermo-responsive ionic compound can be tailored by a suitable selection of cations and anions to approximately tune the phase separation temperature to the room temperature.

In this study, four new classes of ammonium- and phosphonium-based ionic compounds with anions of different hydrophilicity, including benzenesulfonate (BS) and 2-naphthalenesulfonate (NS), were synthesized to examine as a powerful draw solute. Further, the applicability of these ionic compounds as draw solutes for FO was intensively investigated in terms of both the thermo-recovery properties and FO performance, thereby providing fresh ideas and guidance toward the development of prospective draw solutes.

## 2. Experimental

### 2.1. Reagents and instrumentation

Tetrabutylphosphonium bromide, tetrabutylammonium bromide, sodium benzenesulfonate, and sodium 2-naphthalenesulfonate were purchased from Tokyo Chemical Industry Co., Ltd (Tokyo, Japan). Dichloromethane and anhydrous magnesium sulfate were purchased from DaeJung Chemicals & Metals Co. Ltd (Siheung, Republic of Korea). All reagents and solvents were no longer purified when used in synthesis. Distilled water was produced using the Human Power I^+^ Scholar type (Humancorp, Seoul, Republic of Korea).

To identify the synthesized structure, proton nuclear magnetic resonance (^1^H-NMR; Agilent, MR400 DD2) and Fourier transform infrared (FT-IR; Thermo Fisher Scientific, NICOLET iS20) spectroscopy was performed. ^1^H-NMR (400 MHz) spectra were obtained using deuterium oxide (D_2_O). FT-IR spectroscopy was performed under the attenuated total reflection (ATR) mode at wavenumbers ranging from 4000 to 670 cm^−1^. Conductivity measurements were performed using a conductivity meter (METTLER TOLEDO, Seven2Go Pro). An osmometer (KNAUER, SEMI-MICRO OSMOMETER K-7400) uses the freezing point depression method to measure the osmotic pressure of the solution.

For the LCST characterization, the aqueous solution was analyzed by turbidity measurements (*λ* = 650 nm, UV-Vis; EMCLAB Instruments GmbH, EMC-11D-V) coupled with a temperature controller (Misung Scientific. Co., Ltd, TC200P). Also, Fourier Transform-Nuclear Magnetic Resonance Spectrometer (FT-NMR; 600 MHz, JEOL, JNM ECA-600) is used for obtaining temperature-variable ^1^H-NMR spectra. Water and reverse solute fluxes were determined from height differences between the solution levels on either side of a custom-made U-shaped tube before and after the experiment. The value of reverse solute flux was determined from the difference in the conductivity of the feed solution measured by a conductivity meter (METTLER TOLEDO, Seven2Go Pro) before and after the experiment.

### 2.2. Synthesis of tetrabutylammonium benzenesulfonate ([N_4444_][BS]), tetrabutylphosphonium benzenesulfonate ([P_4444_][BS]), tetrabutylammonium 2-naphthalenesulfonate ([N_4444_][NS]), and tetrabutylphosphonium 2-naphthalenesulfonate ([P_4444_][NS])

A mixture of tetrabutylammonium bromide (3.22 g, 10 mmol) and sodium benzenesulfonate (3.60 g, 20 mmol) in a molar ratio of 1 : 2 was dissolved in distilled water (25 mL) in a 250 mL flat-bottom flask, and the mixing was carried out by magnetic stirrer for 24 h at room temperature. The crude product was extracted three times with dichloromethane, washed three times with distilled water, and dried with the addition of anhydrous magnesium sulfate. After filtering out the drying agent, the solvent was eliminated using a rotary evaporator at 50 °C. The concentrated product was dried overnight in a vacuum oven at 80 °C. [N_4444_][BS] was finally obtained, and [P_4444_][BS], [N_4444_][NS], and [P_4444_][NS] were prepared using a synthesis method similar to that applied for the preparation of [N_4444_][BS] (the stoichiometry of the reagents was maintained while varying the amount of added water). [P_4444_][BS] was prepared by dissolving tetrabutylphosphonium bromide (3.39 g, 10 mmol) and sodium benzenesulfonate (3.60 g, 20 mmol) in distilled water (20 mL), [N_4444_][NS] was prepared by dissolving tetrabutylammonium bromide (3.22 g, 10 mmol) and sodium 2-naphthalenesulfonate (4.60 g, 20 mmol) in distilled water (40 mL), and [P_4444_][NS] was prepared by dissolving tetrabutylphosphonium bromide (3.39 g, 10 mmol) and sodium 2-naphthalenesulfonate (4.60 g, 20 mmol) in distilled water (45 mL).

^1^H-NMR of [N_4444_][BS] [400 MHz, D_2_O, *δ*/ppm]: 0.86–0.99 (t, 12H, (C*H*_3_–CH_2_–CH_2_–CH_2_–N^+^–)), 1.26–1.40 (m, 8H, (CH_3_–C*H*_2_–CH_2_–CH_2_–N^+^–)), 1.56–1.70 (m, 8H, (CH_3_–CH_2_–C*H*_2_–CH_2_–N^+^–)), 3.10–3.24 (t, 8H, (CH_3_–CH_2_–CH_2_–C*H*_2_–N^+^–)), 7.49–7.61 (t, 3H, (*Ph*–*H*–SO_3_^−^)), 7.75–7.84 (d, 2H, (*Ph*–*H*–SO_3_^−^)).

^1^H-NMR of [P_4444_][BS] [400 MHz, D_2_O, *δ*/ppm]: 0.75–0.98 (t, 12H, (C*H*_3_–CH_2_–CH_2_–CH_2_–P^+^–)), 1.23–1.62 (m, 16H, (CH_3_–C*H*_2_–C*H*_2_–CH_2_–P^+^–)), 2.10–2.23 (t, 8H, (CH_3_–CH_2_–CH_2_–C*H*_2_–P^+^–)), 7.52–7.75 (t, 3H, (*Ph*–*H*–SO_3_^−^)), 7.75–7.83 (d, 2H, (*Ph*–*H*–SO_3_^−^)).

^1^H-NMR of [N_4444_][NS] [400 MHz, D_2_O, *δ*/ppm]: 0.84–0.96 (t, 12H, (C*H*_3_–CH_2_–CH_2_–CH_2_–N^+^–)), 1.21–1.36 (m, 8H, (CH_3_–C*H*_2_–CH_2_–CH_2_–N^+^–)), 1.46–1.63 (m, 8H, (CH_3_–CH_2_–C*H*_2_–CH_2_–N^+^–)), 3.00–3.15 (t, 8H, (CH_3_–CH_2_–CH_2_–C*H*_2_–N^+^–)), 7.62–7.70 (t, 2H, (*naph*–*H*–SO_3_^−^)), 7.83–7.87 (d, 1H, (*naph*–*H*–SO_3_^−^)), 7.95–8.10 (d, 3H, (*naph*–*H*–SO_3_^−^)), 8.32–8.38 (s, 1H, (*naph*–*H*–SO_3_^−^)).

^1^H-NMR of [P_4444_][NS] [400 MHz, D_2_O, *δ*/ppm]: 0.76–0.98 (t, 12H, (C*H*_3_–CH_2_–CH_2_–CH_2_–P^+^–)), 1.31–1.55 (m, 16H, (CH_3_–C*H*_2_–C*H*_2_–CH_2_–P^+^–)), 1.94–2.16 (t, 8H, (CH_3_–CH_2_–CH_2_–C*H*_2_–P^+^–)), 7.61–7.75 (t, 2H, (*naph*–*H*–SO_3_^−^)), 7.81–7.91 (d, 1H, (*naph*–*H*–SO_3_^−^)), 7.97–8.13 (d, 3H, (*naph*–*H*–SO_3_^−^)), 8.33–8.42 (s, 1H, (*naph*–*H*–SO_3_^−^)).

### 2.3. FO performance

The water flux is an important consideration in the FO process, and it was assessed through the FO test. The FO test was carried out in a custom-designed FO setup that contained U-shaped glass tubes facing L-shaped glass tubes. A semi-permeable membrane (thin-film composite FO membrane (TFC), diameter of 2.06 cm) was developed by Hydration Technologies Inc. (HTI, Albany, Oregon, United States) and was centrally placed in a circular channel between the glass tubes. The entire setup was exposed to air at room temperature. When the system was operated, membrane orientation consist of two modes both the active layer facing the draw solution (AL-DS) and the active layer facing the feed solution (AL-FS). Under the AL-DS mode, one tube was dosed with a draw solution facing the active layer of the membrane, and another tube was dosed with distilled water as the feed solution. Under the AL-FS mode, the active layer of the membrane was placed opposite that in the AL-DS mode. The draw and feed solutions were simultaneously stirred by a magnetic bar using a solenoid (AS ONE, OCTOPUS CS-4) to well blend the existing draw solution and fresh water transferred from the feed solution. The water flux (*J*_w_) was used to quantify the amount of water flowing in direction of the draw solution by measuring the height difference of the draw solution before and after FO. The water flux (*J*_w_, L m^−2^ h^−1^, LMH) was calculated as shown in eqn (1):1
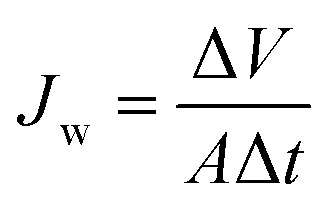
where Δ*V* denotes the change in volume of the draw solution over time (Δ*t*), and *A* represents the effective membrane area (3.32 × 10^−4^ m^2^).

The reverse solute flux (*J*_s_) was obtained by analyzing the quantity of the draw solute that permeated through the FO membrane by means of total dissolved solids (TDS) in the feed solution. In addition, the conversion factor between the TDS (mg L^−1^) and electrical conductivity (μS cm^−1^) was 0.64.^54^ The change in the conductivity and volume of the feed solution before and after FO was measured to calculate the reverse solute flux (*J*_s_, g m^−2^ h^−1^, gMH) using the following eqn (2):2
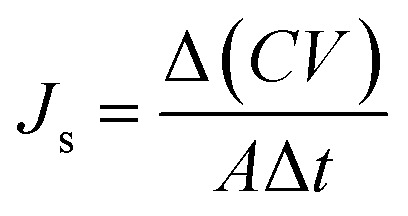
where Δ*C* denotes the change in concentration of the feed solution, Δ*V* represents the volume change, and Δ*t* is time during FO.

## 3. Results and discussion

### 3.1. Synthesis and characterization of [N_4444_][BS], [P_4444_][BS], [N_4444_][NS], and [P_4444_][NS]

**Fig. 1:**
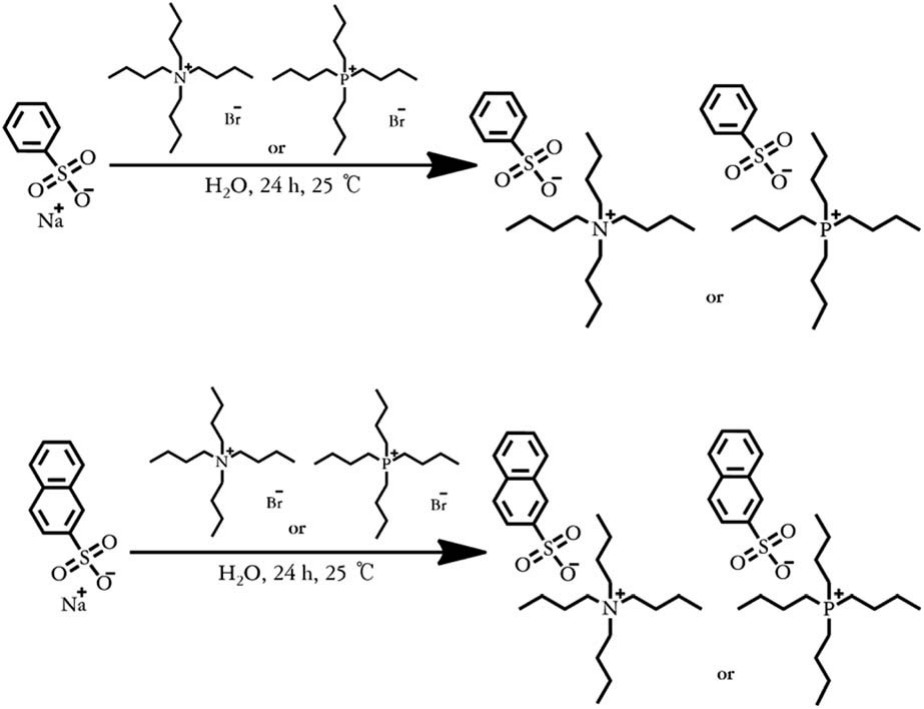
Synthetic scheme of [N_4444_][BS] (top), [P_4444_][BS] (top), [N_4444_][NS] (bottom), and [P_4444_][NS] (bottom).

Fig. 1 illustrates a synthetic scheme of the analogous series, where it can be observed that [N_4444_][BS], [P_4444_][BS], [N_4444_][NS], and [P_4444_][NS] were synthesized *via* an anion exchange reaction of tetrabutylammonium bromide and tetrabutylphosphonium bromide with sodium benzenesulfonate and sodium 2-naphthalenesulfonate. The chemical structures of the synthesized materials were verified by analyzing the ^1^H-NMR and FT-IR data.

**Fig. 2:**
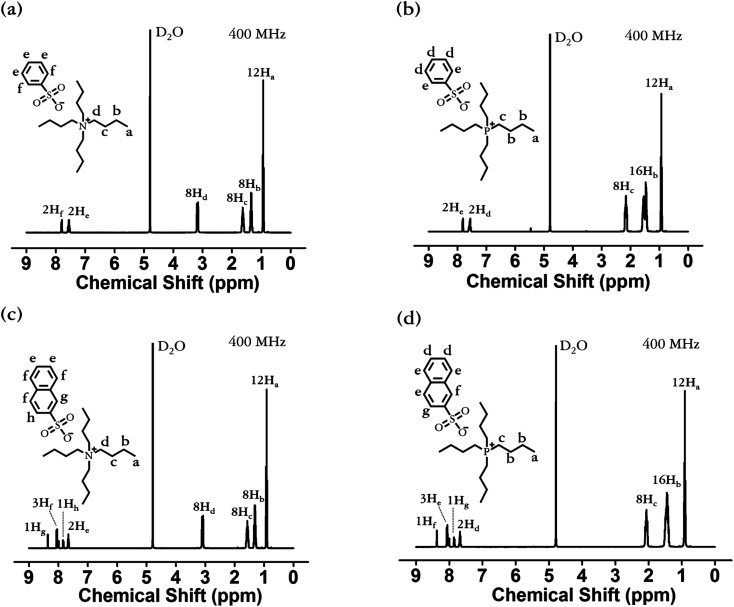
Proton nuclear magnetic resonance (^1^H-NMR) spectra of (a) [N_4444_][BS], (b) [P_4444_][BS], (c) [N_4444_][NS], and (d) [P_4444_][NS].

The ^1^H-NMR spectra of [N_4444_][BS], [P_4444_][BS], [N_4444_][NS], and [P_4444_][NS] are depicted in Fig. 2. The ^1^H-NMR spectra confirm the presence of protons from the alkyl group of [N_4444_]^+^ (*δ* = 0.84–0.99 (peak a), 1.21–1.40 (peak b), 1.46–1.70 (peak c), and 3.00–3.24 ppm (peak d)); [P_4444_]^+^ (*δ* = 0.75–0.98 (peak a), 1.23–1.62 (peak b, c), and 1.94–2.23 (peak d)); the phenyl groups of [BS]^−^ (*δ* = 7.49–7.75 (peak e), and 7.75–7.84 (peak f)); and the naphthyl groups of [NS]^−^ (*δ* = 7.61–7.75 (peak e), 7.81–7.91 (peak h), 7.95–8.13 (peak f), and 8.32–8.42 (peak g)); the integral ratio of each peak is ideally presented as a ratio of the predicted number of hydrogens in each chemical environment.

**Fig. 3:**
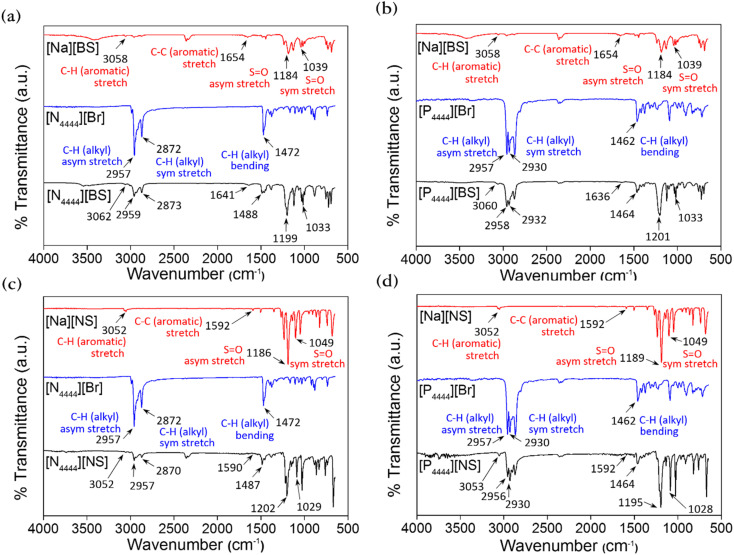
Fourier transform-infrared (FT-IR) spectra of (a) [N_4444_][BS], (b) [P_4444_][BS], (c) [N_4444_][NS], and (d) [P_4444_][NS].

FT-IR spectroscopy was also utilized to confirm that the aforementioned series were synthesized or not, and the FT-IR spectra are presented in Fig. 3. The FT-IR spectra of all materials in this series exhibit the vibrational C–H stretching peak of the aromatic ring at approximately 3052–3062 cm^−1^.^55^ The characteristic peaks related to the C–H stretching vibration and –CH_2_– bending vibration of the alkyl chain can be observed at approximately 2870–2959 cm^−1^ and 1464–1488 cm^−1^, respectively.^56,57^ The vibrational S=O stretching peaks of the sulfonate group appear at approximately 1189–1202 and 1025–1049 cm^−1^, corresponding to asymmetric and symmetric stretching, respectively.^58^ Thus, as a result of the FT-IR analysis, we identified the presence of [N_4444_]^+^, [P_4444_]^+^, [BS]^−^, and [NS]^−^ based on the characteristic IR peaks of functional groups, such as aromatic, sulfonate, and alkyl groups, thereby confirming that [N_4444_][BS], [P_4444_][BS], [N_4444_][NS], and [P_4444_][NS] were successfully synthesized.

### 3.2. Electrical conductivity

Electrical conductivity quantifies the extent to which a material conducts electricity and is mostly affected by the ionic concentrations and type of ions. In addition, small ions tend to demonstrate less resistance and attract polar water molecules more strongly, resulting in freely moving hydrated ions. As the extent of hydration (which depends on the size of the ions) increases, the ionic conductivity is enhanced.^59–61^ Typically, the electrical conductivity tends and the osmotic pressure trend have similarities with respect to the degree of ion generation^62^ The electrical conductivities of all draw solutions whose concentrations are 5, 10, 15, and 20 wt% were measured at room temperature.

**Fig. 4:**
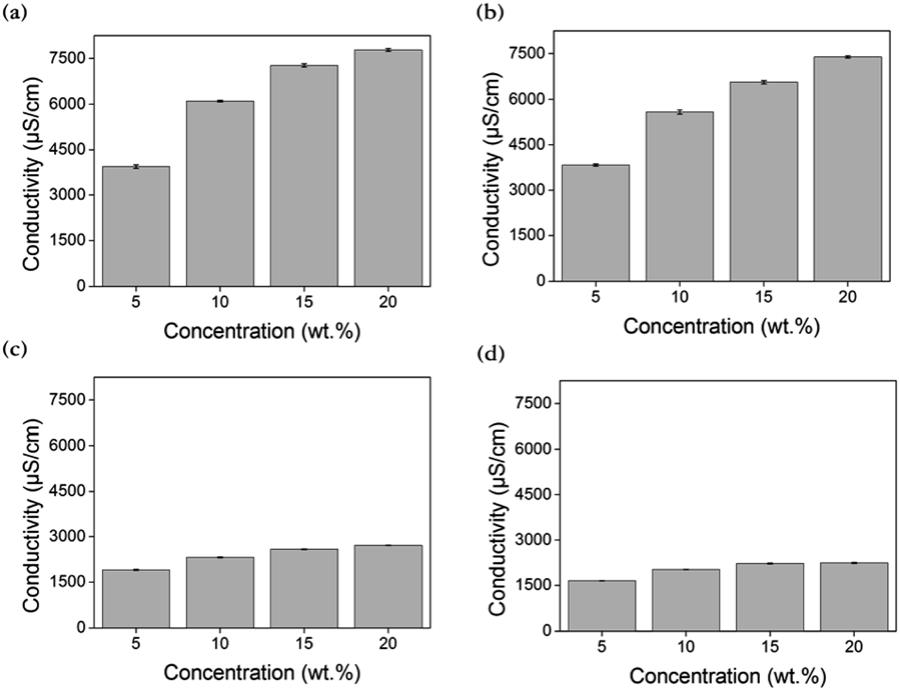
Conductivity of (a) [N_4444_][BS], (b) [P_4444_][BS], (c) [N_4444_][NS], and (d) [P_4444_][NS] according to solution concentration.

According to Fig. 4, the electrical conductivities of [N_4444_][BS], [P_4444_][BS], [N_4444_][NS], and [P_4444_][NS] are approximately 6102, 5581, 2314, and 2025 μS cm^−1^, respectively, when their concentration is 10 wt%. When they have the concentration increased as 20 wt%, the conductivity of [N_4444_][BS], [P_4444_][BS], [N_4444_][NS], and [P_4444_][NS] increases to approximately 7784, 7387, 2714, and 2240 μS cm^−1^, respectively. Fig. 4 indicates that the electrical conductivity was increased following an increase of the concentration of the draw solution. The results in decreasing order of electrical conductivities are as follows: [N_4444_][BS], [P_4444_][BS], [N_4444_][NS], and [P_4444_][NS].

In terms of the structural difference between cations and anions with respect to their size, quaternary ammonium cations and benzene sulfonate anions are smaller than quaternary phosphonium cations and naphthalene sulfonate anions, respectively. Therefore, small ions, that is, quaternary ammonium cations and benzene sulfonate anions induce relatively high electrical conductivities.

### 3.3. Osmotic pressure

During FO, a driving force for water transport into draw solution is the osmotic pressure gradient between the feed and draw solutions. The osmotic pressure is of vital importance in predicting water flux and is mostly relevant for its concentration, which is defined by the van't Hoff equation [eqn (3)], as shown below:^16^

3 *π* = *C*_*i*_*RT*

where *π* denotes the osmotic pressure, *C*_*i*_ represents the molar concentration of solute *i* in the diluted solution, *R* denotes the gas constant, and *T* indicates the absolute temperature of the solution.

**Fig. 5:**
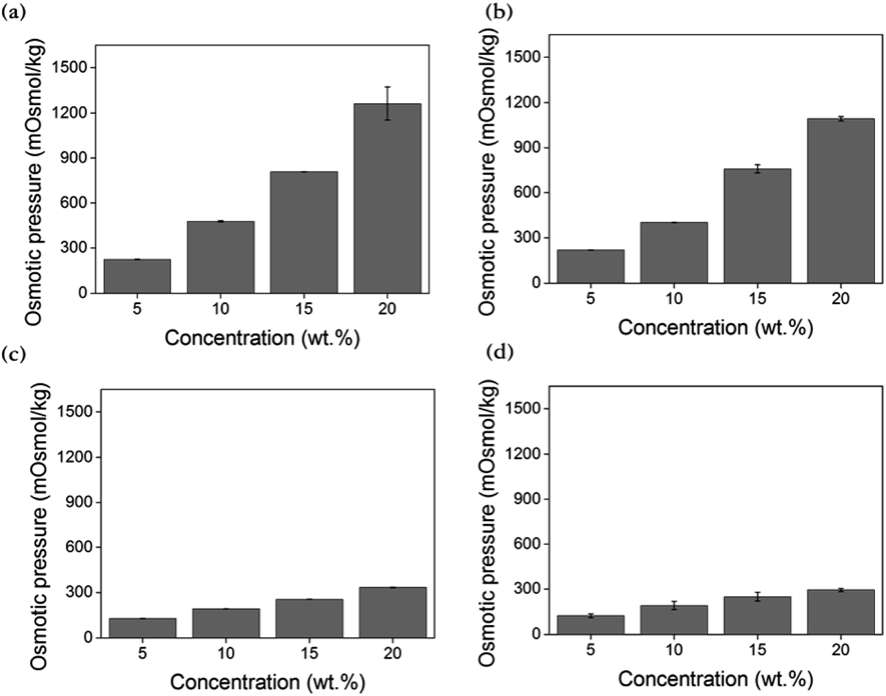
Osmotic pressure of (a) [N_4444_][BS], (b) [P_4444_][BS], (c) [N_4444_][NS], and (d) [P_4444_][NS] according to solution concentration by freezing point depression method.

Through the measurement of osmotic pressure using the freezing point depression method, the applicability of [N_4444_][BS], [P_4444_][BS], [N_4444_][NS], and [P_4444_][NS] as draw solutes was assessed. As presented in Fig. 5, the osmotic pressures of [N_4444_][BS], [P_4444_][BS], [N_4444_][NS], and [P_4444_][NS] are approximately 477, 401, 190, and 189 mOsmol kg^−1^, respectively, at 10 wt%. In addition, the osmotic pressures of [N_4444_][BS], [P_4444_][BS], [N_4444_][NS], and [P_4444_][NS] increase to approximately 1262, 1093, 333, and 294 mOsmol kg^−1^, respectively, when they have the concentration increased as 20 wt%. The osmotic pressure is a colligative property that is inextricably linked with the number of solute particles in the solution and increases with the increasing concentration of the draw solution, as expected. Water solubility and molecular weight play important roles in generating osmotic pressure, which implies that increasing the molecular polarity and decreasing the molecular weight leads to a rise in the osmotic pressure. The osmotic pressures of ionic compounds formed by the quaternary ammonium cations are higher than those of the ionic compounds formed by the quaternary phosphonium cations at the same concentration. This is because the electronegativity difference between nitrogen and carbon is larger than that between phosphorus and carbon.^63^ In addition, the osmotic pressures of ionic compounds that form naphthalene sulfonate anions are lower than those of ionic compounds that form benzene sulfonate anions. This is because the benzene moiety is relatively smaller than the naphthalene moiety, and it is known that a smaller hydrophobic motif increases water solubility. Moreover, solutes with lower molecular weights can generate higher osmotic pressure because they have a significantly higher number of ions than solutes with higher molecular weights at equal masses. Such solutes can induce a relatively higher osmotic pressure than ionic compounds formed by quaternary phosphonium cations or naphthalene sulfonate anions. The compounds in descending order of their osmotic pressures are as follows: [N_4444_][BS], [P_4444_][BS], [N_4444_][NS], and [P_4444_][NS], and this order presents the order consistent with their electrical conductivities.

### 3.4. Recovery properties

**Fig. 6:**
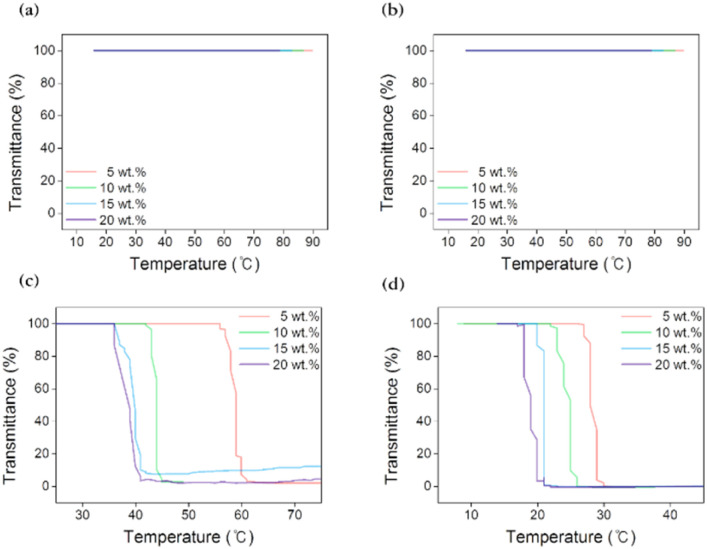
Lower critical solution temperature (LCST) behaviour of (a) [N_4444_][BS], (b) [P_4444_][BS], (c) [N_4444_][NS], and (d) [P_4444_][NS].

In a study on the practical application of the FO process, the main point is whether energy was reduced or not in the recovery step, and thus recovery properties of the draw agent are crucial to the feasibility of FO process. The LCST denotes the phase-transition temperature, and a monophasic and transparent solution is observed below the LCST, whereas a biphasic and opaque solution is observed above the LCST. The LCST can help achieve efficient recovery through thermally triggered transformations to make the separation from the draw solution into draw solutes and pure water. The LCSTs of aqueous [N_4444_][BS], [P_4444_][BS], [N_4444_][NS], and [P_4444_][NS] solution were measured using UV-Vis spectrophotometry in combination with a temperature controller, and temperature point at which the transmittance was 50% was defined as the LCST phase-transition temperature by Fig. 6 presenting transmittance changes according to increase in temperature.

As depicted in Fig. 6, the ionic compounds that form monocyclic aromatics, [N_4444_][BS] and [P_4444_][BS], do not exhibit any changes in their transmittances at 100% while the temperature is controlled from 0 to 100 °C. [N_4444_][BS] and [P_4444_][BS] are extremely hydrophilic; thus, their aqueous solutions undergo a stable homogeneous phase change regardless of the temperature change. This result suggests that [N_4444_][BS] and [P_4444_][BS] are unfit to be applicable as draw solutes when external heat stimuli are used to change the temperature in FO. In contrast, it is interesting to note that ion compounds that form bicyclic aromatics, [N_4444_][NS] and [P_4444_][NS], demonstrate LCST properties. This is because the bulkier naphthyl group has a symmetric structure and is delocalized by intramolecular electron transfer, which favors phase separation.^64^ For example, when the concentrations of the aqueous solutions were 5, 10, 15, and 20 wt%, the LCSTs of [N_4444_][NS] were approximately 59, 44, 39, and 37 °C, respectively. Additionally, the respective LCSTs of [P_4444_][NS] were approximately 28, 25, 21, and 19 °C. Therefore, [N_4444_][NS] and [P_4444_][NS] aqueous solutions could be simply separated from draw solute and pure water by heating and cooling, and they require minimal recovery energy for FO owing to their thermo-responsiveness, whereby LCSTs of those close to room temperature. A comparison of the LCST behavior of [N_4444_]^+^ and [P_4444_]^+^ cations coupled with [NS]^−^ anions indicated that ammonium cations are less hydrophobic than phosphonium cations and interact more *via* water–H bonding.

Thus, ammonium cation-based [N_4444_][NS] was confirmed to have higher phase-transition temperature values than phosphonium cation-based [P_4444_][NS] owing to the high energy requirements of collapsing intensive interactions between the draw solute and water molecules. This behavior is reasonably comprehensible from the viewpoint of the temperature–water affinity relationship of ionic compounds in water. To elaborate further, when the solution temperature is below the LCST, [N_4444_][NS] and [P_4444_][NS] dissolve well in water as they form intensive H-bonds with water molecules. The protons on the alkyl moieties of the quaternary cations of [N_4444_][NS] and [P_4444_][NS] and the aromatic moieties of the anion have the ability to accept the oxygen lone pairs in water.^65^ In addition, when the solution temperature increases above the LCST with gentle heating, the ion–ion interactions, which occur between [N_4444_]^+^ and [NS]^−^ and between [P_4444_]^+^ and [NS]^−^, are more dominant than the ion–water interactions, contributing to the aggregation and subsequently causing turbidity and formation of a heterogeneous solution.

**Fig. 7:**
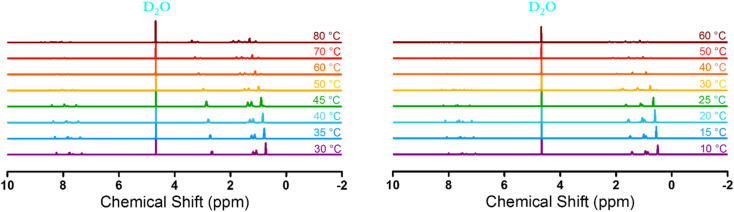
Temperature-variable ^1^H-NMR spectra of [N_4444_][NS] (left) and [P_4444_][NS] (right) from 30 to 80 °C and from 10 to 60 °C, respectively, in D_2_O (15 wt%).

Furthermore, along with turbidity measurement, temperature-variable ^1^H-NMR was measured to quantitatively describe the phase transition degree of [P_4444_][NS] and [N_4444_][NS]. The measurement of temperature-variable ^1^H-NMR was carried out using 15 wt% [P_4444_][NS] and [N_4444_][NS] solution in D_2_O at the temperature range from 10 to 60 °C and from 30 to 80 °C, respectively, in consideration of respective phase transition temperature. Note that all the signals are normalized using the integrated intensity of the solvent (D_2_O) peak as a reference because the solvent peak does not shift during the entire procedure. As depicted in Fig. 7, all peaks corresponding to [P_4444_][NS] and [N_4444_][NS] shift toward a downfield with temperatures increasing. This result indicates that [P_4444_][NS] and [N_4444_][NS] undergo dynamic hydrogen bonds and change in the hydration degree with the increase of temperature.^66–68^ All the intensity of each peak decreases upon increasing the temperature, which seems to suggest that their protons are wrapped due to aggregation after phase transition.^69,70^

### 3.5. Water and reverse solute fluxes

**Fig. 8:**
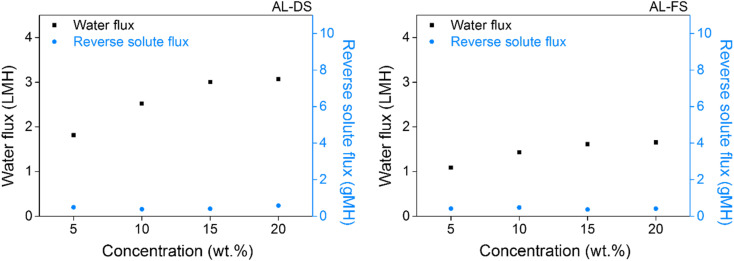
Water and reverse solute flux of [N_4444_][NS] in AL-DS (left) and AL-FS mode (right) during forward osmosis operation at room temperature.

Water and reverse solute fluxes were measured to test the applicability of [N_4444_][NS] as a draw solute. The ionic compounds that exhibited LCST properties in the aforementioned series were [N_4444_][NS] and [P_4444_][NS]. Using [P_4444_][NS] as a draw solute did not contribute to FO efficiencies, which required extra energy consumption. The measurements were carried out at room temperature and it notes that FO process could be operated without supplying additional energy. FO was used for the AL-DS and AL-FS membrane configurations by custom-made glass tubes with the following concentrations of draw solutions: 5, 10, 15, and 20 wt%. As presented in Fig. 8, the water fluxes of [N_4444_][NS] are approximately 1.82, 2.52, 3.01, and 3.07 LMH at 5, 10, 15, and 20 wt% under the AL-DS mode, respectively. When the membrane is inversely used, that is, in the AL-FS mode, the water flux of [N_4444_][NS] decreases to 1.09, 1.43, 1.61, and 1.65 LMH, respectively, at the abovementioned concentrations. The AL-DS mode suffered the less severe effects of dilutive internal concentration polarization (ICP), which reduces the effective osmotic pressure driving force (Δ*π*_eff_) across the membrane. Moreover, concentrative ICP does not increase owing to the use of distilled water as a feed stream in the AL-DS mode. Thus, the overall Δ*π*_eff_ in the AL-DS mode is relatively superior to that in the AL-FS mode, and consequently, exhibits much better water flux values than the AL-FS mode.^71^ When the membrane is used in both two modes, the water permeability of [N_4444_][NS] was increased following an increase of [N_4444_][NS] concentration. The reason for this phenomenon is that the driving force increases coming from an increasing amount of draw solute in solution; hence, the water flux becomes higher too.

Reverse solute flux is an inevitable performance-degrading property that has a negative impact on healthy FO operation. In an ideal semipermeable membrane, the diffusion of draw solute would not occur into the feed solution; however, a realistic membrane would unavoidably lead to movement of draw solute passing through the membrane. An occurrence of reverse solute flux is motivated by the concentration gradient of a draw solute existing between both solution sides, and the corresponding value of [N_4444_][NS] is measured simultaneously to measure the water flux. The reverse solute flux was determined from the TDS of the feed solution, analyzed using the conductivity meter before and after the FO process, which indicates the amount of draw solute that permeates FO membrane. The results indicate that [N_4444_][NS] has a lower propensity to migrate towards the feed solution, and its reverse solute flux is observed to be *ca.* 0.38 and 0.58 gMH and *ca.* 0.37 and 0.48 gMH at concentrations between 5 and 20 wt% under the AL-DS and AL-FS mode, respectively.

## 4. Conclusions

In this study, a series, which is composed of a combination of cations (tetrabutylammonium or tetrabutylphosphonium) and anions (benzenesulfonate or 2-naphthalenesulfonate), was synthesized to examine its applicability as a draw solute in FO. Tetrabutylammonium 2-naphthalenesulfonate ([N_4444_][NS]) and tetrabutylphosphonium 2-naphthalenesulfonate ([P_4444_][NS]) demonstrated LCST characteristics. The LCSTs of [N_4444_][NS] and [P_4444_][NS] were approximately 37 and 19 °C, respectively, at 20 wt%. The recovery temperatures of [N_4444_][NS] and [P_4444_][NS] are close to room temperature. The LCST of [P_4444_][NS] is below room temperature, indicating that [N_4444_][NS] can optimistically serve as a draw solute to make the FO process energy-efficient in this series. Additionally, energy consumption is required, which involves heating to a temperature above the LCST of [P_4444_][NS] or cooling to a temperature below that of [P_4444_][NS], not only to recover the draw solute but also to transport water for the acquisition of freshwater product from the feed solution. In terms of the water flux, [N_4444_][NS] has values of approximately 3.07 LMH and 1.65 LMH at 20 wt% in the AL-DS and AL-FS mode, and in terms of reverse solute flux, it has a value of approximately 0.58 gMH and 0.41 gMH under the same conditions, respectively. In addition, this series, which demonstrated LCST characteristics, improved FO efficiencies due to its characteristic recovery temperature near room temperature, and consequently, the solutes required lower amounts of energy for recovery than other draw solutes. As a result, this research is of help in study of designing and synthesizing thermo-responsive ionic compounds for draw solute application.

## Conflicts of interest

There are no conflicts to declare.

## Supplementary Material
